# Physiological and Metabolic Adaptation to Heat Stress at Different Altitudes in Yaks

**DOI:** 10.3390/metabo12111082

**Published:** 2022-11-08

**Authors:** Shuli Yang, Jinfeng Liu, Zhaobing Gu, Ping Liu, Qin Lan

**Affiliations:** 1 Guangdong Provincial Key Laboratory of Animal Molecular Design and Precise Breeding, College of Life Science and Engineering, Foshan University, Foshan 528231, China; 2 Faculty of Animal Science and Technology, Yunnan Agricultural University, Kunming 650201, China; 3 Yunnan Provincial Key Laboratory of Animal Nutrition and Feed Science, Kunming 650201, China

**Keywords:** yaks, altitudes, heat stress, vasodilatation, metabolic adaptation

## Abstract

Yaks have strong adaptability to extremely cold and hypoxic conditions but are susceptible to high ambient temperature when yaks are raised in low-altitude areas during the high-temperature season. Twenty-four adult male yaks with similar weights and ages were randomly divided into TN (Thermoneutral, altitude = 3464 m), LHS (Light heat stress, altitude = 1960 m), and MHS (Medium heat stress, altitude = 906 m) groups to evaluate adaptation strategies to HS. Non-targeted and targeted metabolomics were applied to investigate the effects of different extents of HS on yaks. LHS- and MHS-yaks showed higher rectal temperatures and respiratory rates than TN-yaks. MHS-yaks had higher levels of red blood cells (RBCs), hemoglobin (Hb), whole blood relative index of middle shear at a shear rate of 5 S^−1^ (WMS), whole blood relative index of high shear at a shear rate of 200 S^−1^ (WHS), Casson viscosity (CV), middle shear flow resistance at a shear rate of 5 S^−1^ (MSFR), and high shear flow resistance at a shear rate of 200 S^−1^ (HSFR) as compared to TN- and LHS-yaks. Differential metabolites and metabolic pathways, including fatty acid metabolism, lipid metabolism, glucose metabolism, and amino acid metabolism, were altered by HS. Metabolites in the glucose metabolism pathway in LHS- and MHS-yaks were lower than those in TN-yaks. However, LHS-yaks showed higher levels of metabolites in the HIF-1 signaling pathway compared to TN- and MHS-yaks. Most of the tricarboxylic acid cycle (TCA) intermediates and fatty acids were significantly decreased in MHS-yaks compared to the other two groups. As a whole, yaks raised at a low altitude (25.6 °C) suffered from severe HS, but they adapted to HS with vasodilatation for dissipating heat and the increased antioxidants and metabolite levels of energy substrates.

## 1. Introduction

There are 20 million yaks (*Bos grunniens*) in the world, of which 90% reside in China [1]. Yaks are mainly distributed in high-altitude regions ranging from 3000 m to 5000 m, and their meat is characterized by high protein and low cholesterol contents. However, a long grass-withering period of about 7–8 months restricts the development of yak husbandry. Moreover, the thermoneutral temperature for yaks is about 5–13 °C, and the optimum temperature is 9 °C [2]. As a unique livestock species, yaks can adapt to the extremely cold climate in the Tibetan Plateau due to genetic evolution under selective pressure, but this is at the cost of decreased growth and reproduction performances. Yaks gain body weight in the warm season but lose ~25% in the cold season [3]. Only 3.7 million yaks are marketed each year out of the 20 million head in stock [1] due to nutrient deficiency resulting from the long grass-withering period and the great energy maintenance requirement in an extremely cold environment [4]. The lactation curve of yaks begins to rise in May, peaks in July, then slowly declines in August before a dramatic decline in September. At high altitudes, yak milk yield and the concentration of unsaturated fatty acids in the cold season are significantly decreased compared to the warm season [5].

Yaks, as one type of seasonal breeder, have a high estrus rate from July to September when the climate is comfortable. Thereafter, the estrus rate of yaks decreases dramatically around November. Female yaks have higher levels of reproductive hormones in the warm season than in the cold season [6], and sperm quality shows the same seasonal variation [7]. Yaks only reproduce 40~60% of the year [8], and they calve once every 2 years or twice in 3 years. Obviously, the cold season has adverse impacts on the growth performance, estrus rate, and mating activity of yaks. Great seasonal variation in production and nutritional value of forage grass between the cold and warm seasons limits the productive performances of yaks [9,10]. The development of yak husbandry in low-altitude areas can make full use of adequate forage to improve economic benefit, but yaks may suffer from heat stress (HS) when they are raised in low-altitude areas during the hot season. However, the skin thickness of yaks can change with age, even in native high-altitude regions, which may improve their ability to adapt to a cold climate.

Thick skin, sparse blood vessels, and low sweat gland density of yaks are conducive to cold adaptation at high altitudes. However, long-term exposure to a hyperthermal environment common at low altitudes and even in its natural habitat at high altitudes may cause HS and oxidative stress [11]. Several recent studies demonstrated that oxidative stress participated in the mechanism of animal stress [12,13]. Some free amino acids (AAs) have antioxidant properties in response to HS. Moreover, free fatty acids (FFAs) and other nutrients are utilized for oxidative phosphorylation to improve the negative energy balance of HS animals. Under HS conditions, the levels of circulating AAs, FFAs, and other nutrients may be altered for thermal adaptation. However, there are few reports on HS in yaks raised at low altitudes in high-temperature seasons. Metabolomics is an important tool to reveal the mechanism of animal HS. Here, non-targeted and targeted metabolomics approaches were applied to reveal metabolic adaptation strategies that yaks employ in response to HS when raised in two low-altitude areas in the hot season. This research aimed to enrich the understanding of the metabolic adaptation of yaks to HS in the hot season and the effective use of forage resources in low-altitude areas.

## 2. Materials and Methods

### 2.1. Materials

Animal experiments were conducted in accordance with the principles of the Animal Welfare Act, issued by the Life Sciences Ethics Committee of Yunnan Agricultural University (202009026). We selected 24 adult male yaks with similar body weights (260 ± 12 kg) and ages (4.6 ± 1.6 years) from an extensive farm in Shangri-La County, Diqing Tibetan autonomous prefecture, Yunnan Province (China) with an average elevation of 3464 m above sea level in August. Yaks were randomly divided into three groups (i.e., 8 yaks per treatment). The yaks in the first group (the TN group) were group-housed in the original farm for 10 months to August of the second year. The second and third groups were transported from the original farm to low-altitude farms in October of the first year. After the two groups were raised for ~10 months at a low altitude (1960 m) with light heat stress (the LHS group) or even lower altitude (906 m) with medium heat stress (the MHS group), the field experiment was performed in the hottest month (August) of the second year and lasted for 3 weeks. Average ambient temperatures in the hottest month are 19.7 °C and 28 °C for the low- and lower-altitude areas, respectively. All yaks were group-housed in similar half-open barns with a loafing area and offered the same rations (containing 12 kg whole-plant corn silages and 2 kg concentrate feeding from a feed mill); the nutrient content of whole-plant corn silage and concentrate feeding are shown in Table 1. The ingredients (%) of concentrate feeding include rapeseed meal 7, cottonseed meal 16, distillers dried grains with solubles (DDGS) 15, extruded soybean 6, soybean meal 33, soybean powder 6, yeast extract powder 0.15, CaHPO_4_ 4.5, limestone 2.9, NaCl 5, guanidineacetic acid 0.45, urea 2, premix 2 (the premix provided the following per kg of diets: VA 500,000 IU, VD_3_ 250,000 IU, VE 1500 IU, Cu 125 mg, Fe 120 mg, Zn 1000 mg, Mn 620 mg, Se 3 mg, I 22 mg, Co 5 mg).

### 2.2. Environmental and Physiological Parameter Measurements

Air temperature and humidity were collected hourly with thermometers (±0.2 °C, Testo 175H1, Testo instruments international trading, Co., Ltd., Shanghai, China) to estimate the temperature-humidity index (THI) according to previously published methods [14]: THI = (1.8 × T + 32) − [(0.55 − 0.0055 × RH) × (1.8 × T − 26)], where T is air temperature (°C), and RH is relative humidity (%).

Yak’s rectal temperature was measured with a veterinary mercury thermometer in a temporary tethered state. Respiration rates (RR) were collected at 08:00, 14:00, and 20:00 h using a mobile phone stopwatch. Benezra’s thermal comfort index (BTCI) is recommended to evaluate the adaptability of cattle to tropical conditions [15]. BTCI = RT/38.33 + RR/23, where RT is the rectal temperature (°C), and RR is the respiration rate. BTCI values > 2 indicated poor adaptability to the environment.

### 2.3. Sample Collection and Preparation

Two mL of whole blood was used for hematological analysis. Blood samples were collected in 10 mL vacutainer tubes containing the chelating agent ethylene diamine tetraacetic acid at 11:00, which were used to obtain plasma by centrifugation for 10 min (1400 *g*, 4 °C). Each aliquot (200 μL) of plasma was stored at −80 °C until UPLC-Q-TOF/MS analysis. All frozen samples were thawed at 4 °C, and 100 μL of each sample was transferred into 2 mL centrifuge tubes. A total of 400 μL of cold methanol (−20 °C) was added to each tube, which was vortexed for 60 s and centrifuged for 10 min (12,000 rpm, 4 °C). Then, each supernatant was transferred into another 2 mL centrifuge tube and dried in a vacuum centrifuge (Eppendorf, 5305, Barkhausenweg, Hamburg, Germany). For LC-MS analysis, the samples were re-dissolved in 150 μL 2-chlorobenzalanine (4 ppm)/80% methanol solution, and the supernatant was filtered through a 0.22 μm membrane to obtain the prepared samples. To monitor the stability and repeatability of instrument analysis, quality control (QC) samples were prepared by pooling 20 μL from each sample. Pooled samples were analyzed together with the other samples. The QC samples were inserted regularly and analyzed every 5 samples.

### 2.4. UHPLC−QTOF-MS Analysis

Chromatographic separation was performed using the Thermo Vanquish system equipped with a 2.1 mm × 100 mm ACQUITY UPLC^®^ HSS T3 1.7 µm column (Waters, Ireland) maintained at 40 °C. The temperature of the autosampler was 8 °C. Gradient elution of analytes was carried out with 0.1% formic acid in water (A2) and 0.1% formic acid in acetonitrile (B2) in ESI positive mode or 5 mM ammonium formate in water (A3) and acetonitrile (B3) in ESI negative mode at a flow rate of 0.25 mL/min. A total of 2 μL of each sample was injected after equilibration. The gradient was 2% B2/B3 (*v*/*v*) for 1 min and was linearly increased to 50% from 1 to 9 min, then increased to 98% from 9 to 12 min before being maintained for 1.5 min, and finally reduced to 2% from 13.5 to 14 min and maintained from 14 to 20 min in 2% B2-positive mode (from 14 to 17 min for 2% B3-negative mode).

The ESI-MSn experiments were executed on the Thermo Q Exactive mass spectrometer with a spray voltage of 3.5 kV and −2.5 kV in positive and negative modes, respectively. The ESI source conditions were set as follows: sheath gas at 30 arbitrary units, auxiliary gas at 10 arbitrary units, and capillary temperature at 325 °C. The instrument was set to acquire over the *m*/*z* range of 81–1000 Da for a full scan at a mass resolution of 70,000. In MS-only acquisition, the instrument was set to acquire over the *m*/*z* range of 60–1000 Da, and the accumulation time for the TOFMS scan was set at 0.20 s/spectrum. The product ion scan was acquired using data-dependent acquisition (DDA) with HCD scans. The normalized collision energy was 30 eV. Unnecessary information in MS/MS spectra was removed with dynamic exclusion.

### 2.5. Data Deconvolution and Processing

ProteoWizard software (v3.0.8789, Palo Alto, CA, USA) was used to convert raw MS data (.wiff scan files) to MzXML files, which were then imported into XCMS software (v3.3.2, San Diego, CA, USA). Mass spectra were processed, which included peak identification, peak filtration, and peak alignment. The following parameters were used: bw = 5, ppm = 15, peakwidth = c(5,30), mzwid = 0.015, mzdiff = 0.01, and method = “centWave”. After normalization to total peak intensity, the processed data were uploaded into MetaboAnalyst software for further analysis. Principal component analysis (PCA) and partial least square discriminant analysis (PLS-DA) were performed for both positive and negative modes after log transformation and Pareto scaling. The OPLS-DA model was established using SIMCA-P 13.0 software. The prediction parameters of the OPLS-DA model are R2X, R2Y, and Q2. R2X and R2Y are the model interpretations of the X and Y matrix, respectively, and Q2 is the prediction ability of the model. The model is more stable and reliable when the three parameters are closer to 1. The predictive ability of the model is better at Q2 > 0.5. Metabolites were considered differential if the Variable Importance in Projection (VIP) scores obtained from the orthogonal partial least-squares discriminant analysis (OPLS-DA) model was greater than 1, the fold change (FC) was greater than 1.5, and the *p*-value was less than 0.05.

Metabolite identification was performed by comparing the accuracy of *m*/*z* values (<30 ppm), and MS/MS spectra were interpreted with Metlin (http://metlin.scripps.edu, (accessed on 15 August 2022)), MoNA (https://mona.fiehnlab.ucdavis.edu//, (accessed on 15 August 2022)), and an in-house database by Bionovogene Company (Suzhou, Jiangsu, China) established with authentic standards in 15 April 2021. The variations within the detected metabolites were calculated by one-way analysis of variance (ANOVA) statistical testing with IBM SPSS statistics 22 software. Post hoc testing was performed to obtain the distinct metabolites that were changed when the ANOVA *p*-values were less than 0.05. Significantly differential metabolites were queried in the KEGG (Kyoto Encyclopedia of Genes and Genomes) signaling pathway database, and published articles were also searched for data relating to global metabolism. Hierarchical clustering analysis of the differential metabolites was performed using the log_2_-transformed expression values and a cutoff of *p* < 0.05.

### 2.6. Metabolite Validation by Targeted Metabolomics

Quantification of differential AAs, fatty acids, and tricarboxylic acid cycle (TCA) intermediates related to HS was performed using liquid chromatography/mass spectrometry (LC/MS) and gas chromatography/mass spectrometry (GC/MS). Metabolites were validated by multiple reaction monitoring mass spectrometry methods. The separation was conducted using an Agilent 1290 Infinity LC (Agilent Technologies, Santa Clara, CA, USA) equipped with a 5500 QTRAP mass spectrometer (AB SCIEX, Framingham, MA, USA). Further details of the targeted metabolomics method are provided as ESI. Metabolite concentrations were determined using the ratio between the metabolite peak area or internal standard peak area and the calibration curve.

## 3. Results

### 3.1. The Impacts of HS on Physiological Parameters

**Figure 1:**
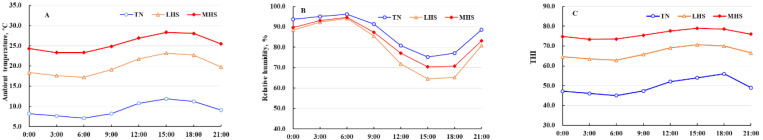
Ambient temperature (**A**), relative humidity (**B**), and temperature humidity indexes (THI) (**C**). TN = Thermal-neutral, LHS = Light heat stress, and MHS = Medium heat stress.

**Figure 2:**
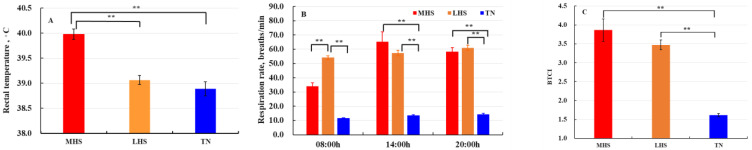
Rectal temperature (**A**), respiration rate (**B**), and Benezra’s thermal comfort index (BTCI) (**C**) of yaks under TN, LHS, and MHS conditions. TN = Thermal-neutral, LHS = Light heat stress, and MHS = Medium heat stress. ** *p* < 0.01.

Air temperature, relative humidity, and THI are shown in Figure 1. The average air temperatures were 9.3 °C, 20.0 °C, and 25.6 °C for the TN, LHS, and MHS regions, respectively. THI values were 49.6, 66.7, and 76.1 for the three yak-raising regions, respectively. The physiological parameters of yaks raised at different altitudes are shown in Figure 2. MHS- and LHS-yaks showed higher BTCI as compared to TN-yaks, and BTCI values of yaks in the two altitude areas are above 2. Respiratory rates of the MHS- and LHS-yaks were significantly greater than that of TN-yaks (*p* < 0.001). MHS-yaks had significantly higher body temperatures (40.0 °C) than that of LHS- and TN-yaks (39.1 °C and 38.9 °C), but no significant differences were detected between the latter two groups.

### 3.2. Hematologic Parameters of Yaks Raised under Different HS Conditions 

MHS-yaks had significantly higher levels of red blood cells (RBCs) and hemoglobin (Hb) as compared to TN- and LHS-yaks, but no differences were detected between TN- and LHS-yaks (Table 2). MHS-yaks had significantly higher levels of WMS, WHS, CV, MSFR, and HSFR than TN-yaks (*p* < 0.05), but no significant differences were detected between LHS- and MHS-yaks.

### 3.3. Metabolic Profiles of Yaks under Different HS Conditions

**Figure 3:**
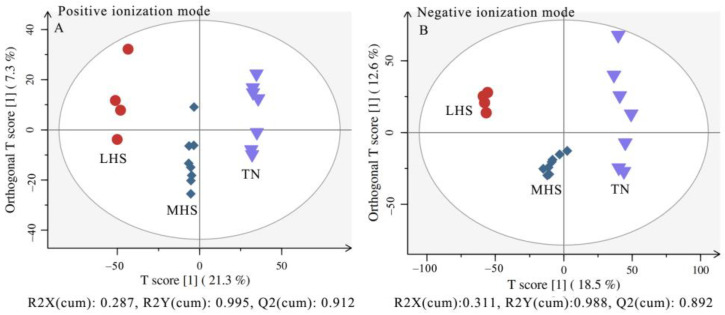
Differentiation of metabolic profiles of yaks under TN, LHS, and MHS conditions using multivariate analysis. (**A**,**B**) OPLS-DA plots of the LC−MS data for the plasma metabolome in positive and negative ionization modes. TN = Thermal-neutral, LHS = Light heat stress, and MHS = Medium heat stress.

Plasma metabolic profiles were further examined to explore the effects of different HS levels of yaks on plasma metabolomics under TN, LHS, and MHS conditions. OPLS-DA plots of the plasma metabolomics data showed an obvious separation of the groups without overlap, indicating that plasma metabolic profiles were distinct. SIMCA-P 13.1 software was used to establish the OPLS-DA model. One predictive component and two orthogonal components were identified with the UHPLC−QTOF-MS metabolomics data. These results indicated that yaks reacted to LHS and MHS with metabolic changes (Figure 3).

### 3.4. Plasma Metabolic Differences of Yaks under Different HS Conditions

A total of 174 metabolites were detected in plasma using a non-targeted analysis. Based on screening criteria of VIP, *p*-value, and fold change (FC), 50 significantly differential metabolites were identified (Table 3, Table 4 and Table 5). These metabolites were further evaluated in the metabolic profiles related to HS.

### 3.5. Characterization and Functional Analysis of Metabolic Pathways

**Figure 4:**
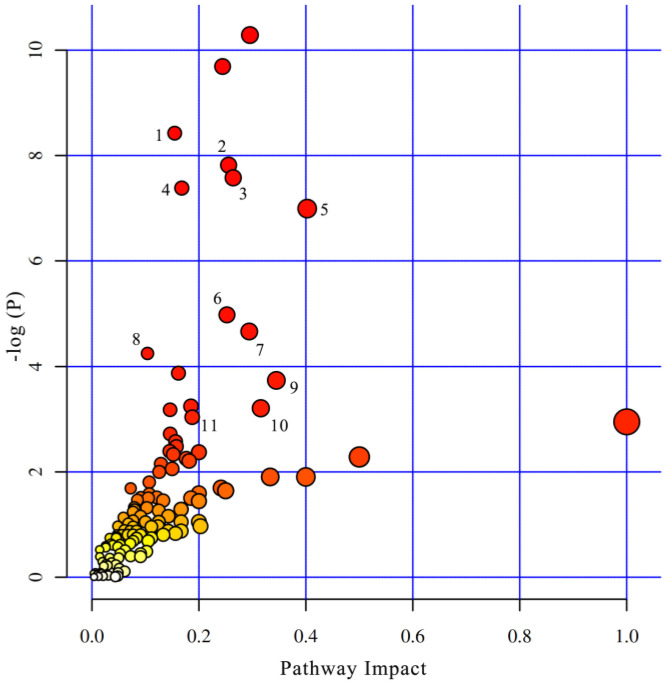
The metabolome-view map of significant metabolic pathways. 1. Phenylalanine metabolism; 2. TCA cycle; 3. Tyrosine metabolism; 4. Arginine biosynthesis; 5. Arginine and proline metabolism; 6. GABAergic synapse; 7. Lysine degradation; 8. Beta-Alanine metabolism; 9. cAMP signaling pathway; 10. HIF-1 signaling pathway; 11. Vascular smooth muscle contraction.

Fourteen metabolic pathways were found when significantly differential metabolites were queried in KEGG, and eleven pathways showed an impact value at the comprehensive level (Table 6). Five types of pathways were revealed: amino acid metabolism, TCA cycle, cAMP signaling, HIF-1 signaling, and vascular smooth muscle contraction (Figure 4). 

### 3.6. Validation of Differential Metabolites with Targeted Metabolomics

**Figure 5:**
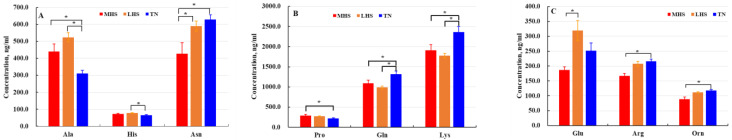
Concentrations of AAs in the plasma of yaks raised under TN, LHS, and MHS conditions. * *p* < 0.05. TN = Thermal-neutral, LHS = Light heat stress, and MHS = Medium heat stress. (**A**–**C**) Concentrations of AAs were validated with targeted metabolomics.

**Figure 6:**
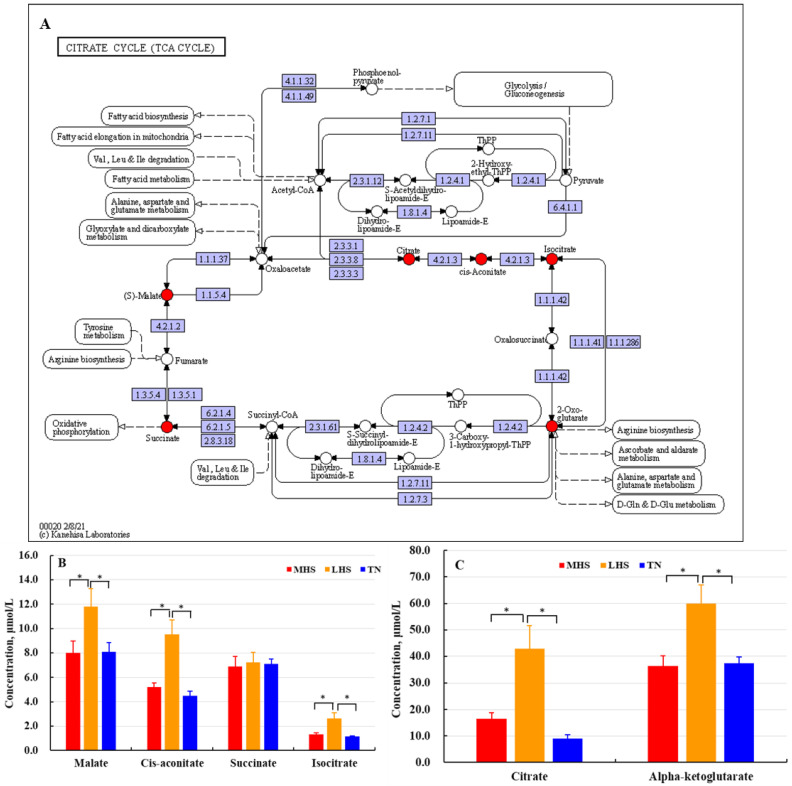
Plasma concentrations of nutrients in yaks under TN, LHS, and MHS conditions. (**A**) The result of non-targeted metabolomics; (**B**,**C**) The validation results of targeted metabolomics. * *p* < 0.05. TN = Thermal-neutral, LHS = Light heat stress, and MHS = Medium heat stress.

Based on the results of non-targeted metabolomics, we validated the differential metabolites with targeted metabolomics. Free amino acid profiles are presented in Table 3. Four of six AAs in LHS- and MHS-yaks occurred at lower levels compared to TN-yaks. Results of validation showed that the level of Ala was significantly higher in the LHS- and MHS-yaks than in the TN-yaks, but MHS-yaks showed lower levels of Asn, Gln, Lys, Arg, and Orn compared to TN-yaks (Figure 5). Similar metabolic profiles for TCA cycle intermediates were detected in non-targeted metabolomics and targeted metabolomics (Table 2, Figure 6). Metabolites in the glucose metabolism pathway were decreased in LHS- and MHS-yaks compared to TN-yaks (Table 2). Results of the validation with targeted metabolomics showed that LHS-yaks had higher levels of saturated fatty acids (SFAs) compared to MHS- and TN-yaks, while no significant differences in most SFAs were detected between the MHS- and TN-yaks (Table 7).

## 4. Discussion

### 4.1. The Impact of HS on Physiological and Hematologic Parameters

We found that MHS-yaks lost their long, thick skirt hair to increase heat loss, but they still showed higher body temperatures and respiratory rates than that of LHS- and TN-yaks, which indicated that yaks raised at lower altitudes (<1000 m) with high ambient temperature suffered severe HS [16]. MHS-yaks had significantly higher levels of RBCs and Hb compared to TN- and LHS-yaks, but previous research reported that HS animals had a high level of Hb but not RBCs [17]. Moderate levels of RBCs and Hb are beneficial to heat dissipation with low blood viscosity and greater blood flow in LHS-yaks. TN-yaks in their native region adapt to hypoxia by increasing the alveolar ventilation rate [18] because high levels of RBCs and Hb may increase blood viscosity and decrease blood flow [19]. 

### 4.2. The Impacts of HS on Vasodilatation for Heat Dissipation

Maximal cutaneous vasodilation induced by HS caused blood flow distribution and hypoxia even in a normoxic environment [20,21]. Compared to TN- and LHS-yaks, MHS-yaks had lower levels of metabolites involved in vasodilation and contraction pathways (Table 4). Metabolites involved in glucose metabolism were decreased in the plasma of MHS-yaks compared to LHS- and TN-yaks. AAs are a kind of macromolecule required in the synthesis of bioactive peptides and proteins and are involved in immune-enhancement, stress alleviation, and other physiological functions [22,23], and we speculated that these AAs are for resistance to HS or oxidative stress.

Ala plays a role in protection against oxidative stress [24], which is conducive to improving the adaptive capacity of yaks to HS. Previous research has speculated that the increase in plasma volume for heat dissipation results in the dilution and low concentrations of most AAs [25]. Arg has immune-enhancing properties [26]; Arg is important in antioxidant protection and associated with the scavenging of excessive free radicals [27,28]. Furthermore, Arg is broken down into nitric oxide by nitric oxide synthases, which stimulates cutaneous vasodilation to improve blood oxygen delivery and heat dissipation from the body surface [29]. Significantly differential vascular smooth muscle contraction pathways were observed between HS- and TN-yaks. Under HS conditions, vasodilation is beneficial to improve cutaneous circulation for the alleviation of HS.

### 4.3. The Impacts of HS on TCA Cycle Intermediates

As a conditionally essential amino acid, Gln is a crucial central point for AA metabolism [30] and a main contributor to immune-enhancement and stress responses [31]. Gln metabolites participate in the TCA cycle, which is an important network of energy metabolism. The concentrations of circulating metabolites of the TCA cycle were decreased in MHS-yaks raised at a very low elevation with high temperature. We detected significantly higher mean levels of TCA cycle intermediates in the LHS group compared to the MHS and TN groups, which may be due to changes in the energy metabolism of HS yaks at a low altitude. Because yaks in the TN group inhabited a thermally comfortable environment in the warm season at a high altitude, their energy expenditure could remain at a low level [32].

### 4.4. The Impacts of HS on Glucose Metabolites and Free Fatty Acids in Plasma

Beyond a function of energy supply, glucose and glucose metabolites are a class of signaling molecules that regulate transcription factors, hormones, and cytokine secretion [33]. Due to low heat increment in fatty acid oxidation compared to starch and fiber utilization [34], fatty acids are the preferred substrate for energy supplementation in HS ruminant animals in a state of negative energy balance [35]. Fatty acids are the main sources of energy in yaks [36]. SFAs, which lack double bonds, can reduce the adverse effects of HS by regulating energy metabolism in mitochondria [37]. Monounsaturated fatty acids (MUFAs), which have a single double bond, are resistant to peroxidative damage [38]. MUFAs were significantly decreased by HS, and the concentrations of 4 of 6 plasma MUFAs were decreased in LHS- and MHS-yaks compared to TN-yaks. Polyunsaturated fatty acids (PUFAs) are more susceptible to free radical-induced damage, but higher levels of most circulating PUFAs were detected in TN- and LHS-yaks compared to MHS-yaks, which is consistent with a previous report [39]. High levels of fatty acids can protect against oxidative stress [40]. Meanwhile, fatty acids, including most SFAs, MUFAs, and PUFAs, were decreased in MHS-yaks by the low-altitude, extremely warm site, which indicated that MHS-yaks are in negative energy balance status under severe HS conditions.

### 4.5. The Impacts of HS on Changes in Plasma Antioxidants

Animals increase their levels of antioxidants for scavenging excessive free radicals under HS. Previous research reported that moderate HS could increase antioxidant production in bacteria [41], but antioxidant production or activity in homothermal animals may be restrained by severe HS. MHS-yaks had lower levels of non-enzymatic antioxidants compared to TN- and LHS-yaks. We also found that the levels of partial vitamins and vitamin analogs, including nicotinic acid, biotin, 4-pyridoxic acid, retinol, and retinoyl β-glucuronide, were decreased in MHS-yaks. Biotin is a member of the vitamin B complex and is an essential coenzyme with anti-oxidation effects that promote the removal of HS- or oxidative stress-induced excess free radicals. The suitable supplementation of biotin can alleviate negative HS in quail [42]. A study has previously reported that skin temperatures of dairy cows were decreased by the supplementation of nicotinic acid through the promotion of heat loss by vasodilatation [43]. We also found that HS resulted in a reduction in vitamin synthesis and production. Stress conditions can cause oxidative stresses [44], and we detected some differential metabolic pathways related to oxidative stress induced by HS. Due to good adaptability to a warm climate for yaks, LHS-area is suitable for the development of the yak industry with rich forage resources. Our research findings may be beneficial to the effective use of forage resources and environmental management to reduce the response to HS of yaks in low-altitude areas. In order to avoid the effect of the ration on plasma metabolites, we provided yaks with the same ration. Due to different levels of oxygen saturation at different altitudes, the evaluation of heat stress was performed when yaks were raised for a 10-month adaptation period to mitigate the effects of oxygen content on plasma metabolites. Moreover, further research is needed to detect the metabolic adaptation strategy of yaks to LHS as compared to their counterparts under thermal-neutral conditions in the same low-altitude area.

## 5. Conclusions

Yaks raised at a very low altitude (<1000 m, 25.6 °C) in August suffered from severe HS as compared to TN- (native area at high altitude-3464 m, 9.3 °C) and LHS-yaks at a low altitude (906 m, 20 °C). Yaks adapt to HS with vasodilatation for dissipating heat and increasing metabolite levels of antioxidants and energy substrates. As a whole, yaks at low altitudes displayed good adaptability to LHS, but they suffered from heat stress at lower altitude areas. Unless a comfortable thermal environment or special feed additives are used to mitigate heat stress, lower altitude area with high temperatures in the hot season are unsuitable for the development of yak husbandry.

## Figures and Tables

**Table 1:** Nutrient content of feed samples (g/kg absolute dry matter intake).

Items	Whole-Plant Corn Silage	Concentrate Feeding
EE	16.8	45.3
Crude ash	64.9	233.3
CP	85.9	439.9
Ca	3.4	36.0
P	2.273	11.8
ADF	400.6	169.5
ADICP	4.980	44.4
NDF	6278.0	225.8
NDICP	2.7	67.7
ADL	56.3	47.5
NFC	343.6	218.1
tdNFC	336.7	213.7
tdNDF	351.9	34.5
tdCP	81.3	389.6
tdFA	5.5	34.4

Abbreviations: EE ether extract, CP crude protein, Ca calcium, P phosphorus, ADF acid detergent fiber, ADICP acid detergent insoluble crude protein, NDF neutral detergent fiber, NDICP neutral detergent insoluble crude protein, ADL acid detergent lignin, NFC non-fiber carbohydrate, tdNDF truly digestible neutral detergent fiber, tdNFC truly digestible non-fiber carbohydrate, tdCP truly digestible crude protein, tdFA truly digestible fatty acids.

**Table 2:** Hematologic parameters of yaks under TN, LHS, and MHS conditions.

Items	MHS	LHS	TN
RBCs	9.1 ± 0.4 ^a^	7.1 ± 0.3 ^b^	7.7 ± 0.5 ^b^
Hb	160.3 ± 7.7 ^a^	123.9 ± 5.5 ^b^	123.8 ± 8.3 ^b^
HCT	52.3 ± 2.5 ^a^	42.0 ± 1.7 ^b^	40.3 ± 2.9 ^b^
MCH	17.6 ± 0.3 ^a^	17.6 ± 0.3 ^a^	16.1 ± 0.5 ^b^
MCHC	306.0 ± 3.7 ^a^	294.6 ± 2.4 ^b^	307.5 ± 2.7 ^a^
RDW	15.1 ± 0.1 ^b^	15.7 ± 0.2 ^a^	14.8 ± 0.1 ^b^
PLT	197.3 ± 25.5	246.3 ± 29.2	207.9 ± 14.7
MCV	57.7 ± 0.5 ^a^	59.8 ± 1.3 ^a^	52.7 ± 2.1 ^b^
MPV	6.5 ± 0.2 ^b^	7.2 ± 0.3 ^a^	6.1 ± 0.2 ^b^
PDW	15.1 ± 0.1	15.7 ± 0.2	14.8 ± 0.1
PCT	0.1 ± 0.0 ^b^	0.2 ± 0.0 ^a^	0.1 ± 0.0 ^b^
Fibrinogen	3.6 ± 0.1	3.6 ± 0.1	3.6 ± 0.1
Blood viscosity	1.6	1.7	1.6
WLS	14.9 ± 0.7	13.2 ± 0.6	12.8 ± 0.9
WMS	3.9 ± 0.2 ^a^	3.4 ± 0.2 ^ab^	3.2 ± 0.2 ^b^
WHS	3.0 ± 0.2 ^a^	2.5 ± 0.1 ^ab^	2.3 ± 0.2 ^b^
EAIRBC	5.0 ± 0.2	5.3 ± 0.3	5.5 ± 0.1
EACRBC	3.4 ± 0.1	3.6 ± 0.2	3.8 ± 0.1
CV	4.1 ± 0.3 ^a^	3.4 ± 0.2 ^ab^	3.1 ± 0.3 ^b^
LSFR	73.4 ± 4.4	65.1 ± 2.7	62.3 ± 5.2
MSFR	45.5 ± 3.3 ^a^	39.0 ± 1.6 ^ab^	35.9 ± 2.9 ^b^
HSFR	34.5 ± 2.7 ^a^	29.2 ± 1.5 ^ab^	26.6 ± 2.2 ^b^

Abbreviations: Hb hemoglobin, RBC red blood cell count, HCT hematocrit, MCV mean corpuscular volume, MCH mean corpuscular hemoglobin, MCHC mean corpuscular hemoglobin concentration, RDW red blood cell distribution width, WLS whole blood relative index of low shear at a shear rate of 1 S^−1^, WMS whole blood relative index of middle shear at a shear rate of 5 S^−1^, WHS whole blood relative index of high shear at a shear rate of 200 S^−1^, EAIRBC aggregation index, EACRBC aggregation coefficient, CV Casson viscosity, EAI RBC aggregation index, EAC RBC aggregation coefficient, LSFR low shear flow resistance at a shear rate of 1 S^−1^, MSFR middle shear flow resistance at a shear rate of 5 S^−1^, HSFR high shear flow resistance at a shear rate of 200 S^−1^. Row values with different lowercase superscripts (a, b) are significantly different at *p* < 0.05. TN = Thermal-neutral, LHS = Light heat stress, and MHS = Medium heat stress.

**Table 3:** Distinct plasma metabolites from three carbohydrate metabolism pathways.

Metabolites	MS2 Values of Metabolites			Fold Change		
	MHS	LHS	TN	MHS/LHS	LHS/TN	MHS/TN
Glucose metabolism pathway						
Beta-D-Glucose	175,503.7 ^b^	49,931.1 ^b^	3,071,592.9 ^a^	3.51	0.02	0.06
6-Phosphogluconic acid	18,480,403.0 ^b^	12,054,071.0 ^c^	25,856,213.7 ^a^	1.53	0.47	0.71
Glucosamine 6-phosphate	1,030,375.5 ^b^	1,603,223.7 ^b^	5,548,853.0 ^a^	0.64	0.29	0.19
HIF-1 signaling pathway						
Pyruvic acid	15,474,148.6 ^b^	36,698,657.4 ^a^	3,491,288.2 ^b^	0.42	10.51	4.43
2-Oxoglutarate	22,091,070.9 ^b^	63,760,679.5 ^a^	23,489,928.1 ^b^	0.35	2.71	0.94
L-Lactic acid	32,899,048.6 ^b^	58,121,217.4 ^a^	28,845,529.4 ^b^	0.57	2.01	1.14
TCA cycle pathway						
Fumaric acid	6,211,170.6 ^b^	4,683,750.2 ^b^	11,401,787.1 ^a^	1.33	0.41	0.54
Citric acid	1,726,608.8 ^b^	3,364,146.0 ^a^	738,279.3 ^c^	0.51	4.56	2.34
Succinic acid	6,623,535.0 ^ab^	9,851,484.2 ^a^	3,873,301.5 ^b^	0.67	2.54	1.71
Isocitric acid	2,976,933.3 ^a^	9,231,321.3 ^a^	389,702.6 ^b^	0.32	23.69	7.64

Data are the MS2 peak area of metabolites. ANOVA statistical test was used to acquire distinct metabolites. Row values with different lowercase superscripts (a, b, c) are significantly different at *p* < 0.05. TN = Thermal-neutral, LHS = Light heat stress, and MHS = Medium heat stress.

**Table 4:** Distinct plasma metabolites of fatty acids, amino acids, and vitamins.

Metabolites	MS2 Values of Metabolites			Fold Change		
	MHS	LHS	TN	MHS/LHS	LHS/TN	MHS/TN
Fatty acid metabolism pathway						
Pelargonic acid	2,229,117.0 ^b^	2,213,080.9 ^b^	2,848,879.6 ^a^	1.01	0.78	0.78
Stearidonic acid	1,067,560.6 ^b^	2,635,754.0 ^b^	49,400,885.4 ^a^	0.41	0.05	0.02
(9Z,12Z,15Z)-Octadecatrienoic acid	2,771,981.0 ^b^	1,914,927.1 ^b^	19,005,417.2 ^a^	1.45	0.10	0.15
12-Hydroxydodecanoic acid	7,278,600.5 ^b^	707,628.0 ^b^	31,411,741.6 ^a^	10.29	0.02	0.23
13S-hydroxyoctadecadienoic acid	2,119,405.4 ^b^	5,815,579.2 ^b^	20,305,676.9 ^a^	0.36	0.29	0.10
Myristic acid	21,186,183.2 ^a^	15,377,109.1 ^b^	23,628,349.1 ^a^	1.38	0.65	0.90
AAs metabolism pathway						
Ornithine	16,400,508.3 ^b^	19,996,688.2 ^ab^	34,191,126.9 ^a^	0.82	0.58	0.48
Tryptophanol	637,479.6 ^b^	315,211.8 ^b^	1,906,959.0 ^a^	2.02	0.17	0.33
S-Adenosylmethionine	563,307,223.8 ^ab^	348,765,603.6 ^b^	739,305,208.6 ^a^	1.62	0.47	0.76
Citrulline	43,367,716.4 ^ab^	28,442,760.6 ^b^	48,783,155.9 ^a^	1.52	0.58	0.89
L-Histidine	51,553,230.9 ^b^	84,089,005.2 ^a^	47,407,454.9 ^b^	0.61	1.77	1.09
N-Acetylglutamic acid	9,402,625.7 ^a^	8,953,631.9 ^ab^	5,142,373.1 ^b^	1.05	1.74	1.83
Vitamin metabolism pathway						
Biotin	1,299,581.1 ^b^	4,590,534.0 ^a^	633,710.7 ^b^	0.28	7.24	2.05
Nicotinic acid	7,388,364.3 ^b^	15,024,678.9 ^a^	2,966,459.0 ^b^	0.49	5.06	2.49
4-Pyridoxic acid	4,177,648.8 ^b^	11,819,506.7 ^a^	4,403,432.5 ^b^	0.35	2.68	0.95
Retinol	2,097,255.4 ^ab^	713,799.2 ^b^	3,137,850.1 ^a^	2.94	0.23	0.67
Retinoyl β-glucuronide	13,269,918.9 ^b^	7,298,404.9 ^b^	36,852,011.4 ^a^	1.82	0.20	0.36

Data are the MS2 peak area of metabolites. ANOVA statistical test was used to acquire distinct metabolites. Row values with different lowercase superscripts (a, b) are significantly different at *p* < 0.05. TN = Thermal-neutral, LHS = Light heat stress, and MHS = Medium heat stress.

**Table 5:** Distinct plasma metabolites relating to non-enzymatic antioxidants and vasodilation.

Metabolites	MS2 Values of Metabolites			Fold Change		
	MHS	LHS	TN	MHS/LHS	LHS/TN	MHS/TN
Non-enzymatic antioxidants pathway						
gamma-Glutamylalanine	5,800,356.4 ^b^	13,433,201.2 ^a^	4,691,901.8 ^b^	0.43	2.86	1.24
Acetylcysteine	17,283,582.8 ^b^	22,770,228.3 ^b^	50,044,587.9 ^a^	0.76	0.45	0.35
N-Acetyl-D-glucosamine	2,791,861.2 ^b^	2,957,486.1 ^ab^	18,595,163.8 ^a^	0.94	0.16	0.15
S-Allylcysteine	1,438,037.1 ^b^	3,211,873.7 ^a^	356,295.7 ^c^	0.45	9.01	4.04
Cyclohexylamine	4,076,873.0 ^b^	12,405,377.7 ^a^	6,227,572.8 ^b^	0.33	1.99	0.65
Diphenylamine	21,728,135.9 ^b^	37,733,206.6 ^a^	15,903,247.7 ^b^	0.58	2.37	1.37
beta-Alanine	21,012,859.0 ^b^	13,131,245.5 ^b^	32,777,332.1 ^a^	1.60	0.40	0.64
4-Hydroxybenzoic acid	4,248,079.2 ^b^	1,307,664.2 ^b^	31,650,555.5 ^a^	3.25	0.04	0.13
Lipoxin A4	31,156,376.5 ^ab^	9,382,760.9 ^b^	55,572,174.5 ^a^	3.32	0.17	0.56
Alantolactone	2,828,685.7 ^a^	2,065,247.4 ^a^	197,593.5 ^b^	1.37	10.45	14.32
Dimethyl sulfone	698,601,726.1 ^a^	631,233,853.5 ^ab^	503,364,423.7 ^b^	1.11	1.25	1.39
Astragalin	27,480,169.6 ^ab^	33,578,853.3 ^a^	24,076,039.9 ^b^	0.82	1.39	1.14
Carnosine	20,248,888.8 ^ab^	27,761,540.4 ^a^	12,088,992.3 ^b^	0.73	2.30	1.67
Vasodilation and contraction pathway						
20-HETE	521,749.1 ^b^	7,614,931.8 ^a^	174,935.9 ^b^	0.07	43.53	2.98
cAMP	11,570,459.4 ^b^	40,205,475.0 ^a^	22,068,413.5 ^ab^	0.29	1.82	0.52
Norepinephrine	121,214,550.9 ^b^	202,047,064.8 ^a^	93,772,652.5 ^b^	0.60	2.15	1.29
L-Homophenylalanine	4,522,816.0 ^b^	9,491,670.1 ^a^	3,241,494.2 ^b^	0.48	2.93	1.40
Lipoxin B4	1,820,822.8 ^b^	6,808,483.6 ^a^	228,616.3 ^b^	0.27	29.78	7.96
1-Methylhistidine	67,795,370.1 ^b^	80,489,665.4 ^a^	27,915,459.0 ^b^	0.84	2.88	2.43
Phenylacetylglycine	202,354,294.8 ^b^	250,158,251.4 ^b^	585,414,282.4 ^a^	0.81	0.43	0.35
Acetylcholine chloride	170,878,035.5 ^b^	158,994,744.4 ^b^	218,408,026.2 ^a^	1.07	0.73	0.78
Coumarin	17,001,752.1 ^b^	19,844,404.4 ^b^	37,670,336.8 ^a^	0.86	0.53	0.45
Prostaglandin F1a	957,098.6 ^b^	900,009.1 ^b^	4,423,172.6 ^a^	1.06	0.20	0.22
Hordenine	2,485,240.9 ^a^	2,066,988.5 ^ab^	1,137,298.6 ^b^	1.20	1.82	2.19
PGA1	2,701,769.3 ^ab^	3,810,276.5 ^a^	1,557,899.4 ^b^	0.71	2.45	1.73

Data are the MS2 peak area of metabolites. ANOVA statistical test was used to acquire distinct metabolites. Row values with different lowercase superscripts (a, b, c) are significantly different at *p* < 0.05. TN = Thermal-neutral, LHS = Light heat stress, and MHS = Medium heat stress.

**Table 6:** KEGG pathway enrichment analysis of differential metabolites in yaks.

Items	Hits ^a^	*p*-Value	Holm *p* ^b^	Impact Value
Phenylalanine metabolism	11	0.000	0.059	0.155
Citrate cycle (TCA cycle)	6	0.000	0.108	0.256
Tyrosine metabolism	12	0.001	0.166	0.168
Arginine biosynthesis	6	0.001	0.245	0.403
Arginine and proline metabolism	10	0.01	1	0.252
GABAergic synapse	3	0.009	1	0.294
Lysine degradation	7	0.014	1	0.104
Beta-Alanine metabolism	5	0.024	1	0.345
cAMP signaling pathway	4	0.039	1	0.185
HIF-1 signaling pathway	3	0.041	1	0.316
Vascular smooth muscle contraction	3	0.048	1	0.187

^a^ Hits represents the number of metabolites in one pathway. ^b^ Holm *p* indicates the statistical *p*-values that were further adjusted using the Holm–Bonferroni method for multiple tests. TN = Thermal-neutral, LHS = Light heat stress, and MHS = Medium heat stress.

**Table 7:** Validated plasma metabolites associated with the adaptation to HS, μg/mL.

Items	Fatty Acids	MHS	LHS	TN
Saturated fatty acids	C6:0	0.01 ± 0.001 ^b^	0.012 ± 0.0 ^a^	0.008 ± 0.001 ^c^
	C8:0	0.001 ± 0.0 ^b^	0.003 ± 0.0 ^a^	0.001 ± 0.0 ^b^
	C10:0	0.003 ± 0.001 ^b^	0.006 ± 0.0 ^a^	0.002 ± 0.0 ^b^
	C16:0	10.018 ± 0.79 ^ab^	12.624 ± 1.159 ^a^	9.091 ± 0.599 ^b^
	C18:0	10.149 ± 1.365 ^b^	15.051 ± 1.261 ^a^	8.276 ± 0.683 ^b^
	C23:0	0.036 ± 0.006 ^ab^	0.026 ± 0.001 ^b^	0.051 ± 0.005 ^a^
Monounsaturated fatty acids	C15:1	0.247 ± 0.016 ^b^	0.217 ± 0.033 ^b^	0.482 ± 0.039 ^a^
	C17:1T	0.122 ± 0.007 ^b^	0.134 ±0.006 ^b^	0.195 ± 0.016 ^a^
	C18:1N9T	0.081 ± 0.012 ^b^	0.112 ± 0.009 ^a^	0.060 ± 0.003 ^b^
	C18:1N12	1.709 ± 0.266 ^a^	1.709 ± 0.176 ^a^	0.662 ± 0.117 ^b^
	C20:1	0.19 ± 0.01 ^b^	0.205 ± 0.014 ^b^	0.418 ± 0.026 ^a^
	C24:1	0.109 ± 0.013 ^ab^	0.089 ± 0.016 ^b^	0.142 ± 0.005 ^a^
Polyunsaturated fatty acids	C18:2N6T	0.021 ± 0.004 ^bc^	0.030 ± 0.002 ^a^	0.022 ± 0.002 ^ab^
	C18:2N6	3.120 ± 1.023 ^b^	7.281 ± 0.508 ^a^	3.341 ± 0.474 ^b^
	C18:3N6	0.159 ± 0.055 ^ab^	0.260 ± 0.019 ^a^	0.082 ± 0.028 ^b^
	C18:3N3	0.260 ± 0.049 ^b^	0.212 ± 0.021 ^b^	1.166 ± 0.082 ^a^
	C20:3N3	0.220 ± 0.035 ^ab^	0.234 ± 0.030 ^a^	0.132 ± 0.025 ^b^
	C22:2	0.033 ± 0.002 ^ab^	0.029 ± 0.003 ^b^	0.040 ± 0.003 ^a^
	C20:5N3	0.095 ± 0.018 ^b^	0.054 ± 0.007 ^b^	0.295 ± 0.058 ^a^
	C22:4	0.121 ± 0.050 ^ab^	0.191 ± 0.012 ^a^	0.035 ± 0.003 ^b^
	C22:5N3	0.240 ± 0.043 ^ab^	0.149 ± 0.015 ^b^	0.364 ± 0.057 ^a^
	C22:6N3	0.108 ± 0.02 ^ab^	0.058 ± 0.008 ^b^	0.128 ± 0.022 ^a^

Abbreviation: C6:0 Caproate, C8:0 Caprylate, C10:0 Caprate, C16:0 Palmitate, C18:0 Stearate, C23:0 Tricosanoate, C15:1 10-Pentadecenoate, C17:1T 10-Transsheptadecenoate, C18:1N9T Elaidate, C18:1N12 Petroselinate, C20:1 11-Eicosenoate, C24:1 Nervonoate, C18:2N6T Linoelaidate, C18:2N6 Linoleate, C18:3N6 Gamma Linolenate, C18:3N3, Alpha Linolenate, C20:3N3, 11-14-17 Eicosatrienoate, C22:2 Docosadienoate, C20:5N3 Eicosapentaenoate, C22:4 Docosatetraenoate, C22:5N3 Docosapentaenoate, and C22:6N3 Docosahexaenoate. Row values with different lowercase superscripts (a, b, c) are significantly different at *p* < 0.05. TN = Thermal-neutral, LHS = Light heat stress, and MHS = Medium heat stress.

## Data Availability

No new data were created or analyzed in this study. Data sharing is not applicable to this article.
